# Schizophrenia Spectrum Disorders Show Reduced Specificity and Less Positive Events in Mental Time Travel

**DOI:** 10.3389/fpsyg.2016.01121

**Published:** 2016-07-26

**Authors:** Xing-jie Chen, Lu-lu Liu, Ji-fang Cui, Ya Wang, An-tao Chen, Feng-hua Li, Wei-hong Wang, Han-feng Zheng, Ming-yuan Gan, Chun-qiu Li, David H. K. Shum, Raymond C. K. Chan

**Affiliations:** ^1^Neuropsychology and Applied Cognitive Neuroscience Laboratory, Key Laboratory of Mental Health, Institute of Psychology, Chinese Academy of SciencesBeijing, China; ^2^College of Humanities, University of Chinese Academy of SciencesBeijing, China; ^3^Department of Psychological and Brain Sciences, University of Massachusetts Amherst, AmherstMA, USA; ^4^Information Center, National Institute of Education SciencesBeijing, China; ^5^School of Psychology, Southwest UniversityChongqing, China; ^6^Beibei Mental Health CenterChongqing, China; ^7^Beijing Huilongguan HospitalBeijing, China; ^8^Menzies Health Institute Queensland and School of Applied Psychology, Griffith University, Gold CoastQLD, Australia

**Keywords:** mental time travel, episodic future thinking, past, schizophrenia, schizophrenia spectrum

## Abstract

Mental time travel refers to the ability to recall past events and to imagine possible future events. Schizophrenia (SCZ) patients have problems in remembering specific personal experiences in the past and imagining what will happen in the future. This study aimed to examine episodic past and future thinking in SCZ spectrum disorders including SCZ patients and individuals with schizotypal personality disorder (SPD) proneness who are at risk for developing SCZ. Thirty-two SCZ patients, 30 SPD proneness individuals, and 33 healthy controls participated in the study. The Sentence Completion for Events from the Past Test (SCEPT) and the Sentence Completion for Events in the Future Test were used to measure past and future thinking abilities. Results showed that SCZ patients showed significantly reduced specificity in recalling past and imagining future events, they generated less proportion of specific and extended events compared to healthy controls. SPD proneness individuals only generated less extended events compared to healthy controls. The reduced specificity was mainly manifested in imagining future events. Both SCZ patients and SPD proneness individuals generated less positive events than controls. These results suggest that mental time travel impairments in SCZ spectrum disorders and have implications for understanding their cognitive and emotional deficits.

## Introduction

Mental time travel refers to the ability to recall autobiographical past events and imagine possible future events (Arzy et al., 2008; Botzung et al., 2008). It is a kind of subjective consciousness about time that ranges from the past to the future (Suddendorf and Corballis, 2007). Thinking about the future and remembering the past are important abilities in maintaining one’ sense of self in time (Atance and O’NeIll, 2001; Tulving et al., 2005). According to the episodic simulation hypothesis (Schacter and Addis, 2007b; Schacter et al., 2007), these two abilities share the same memory system because imagining the future requires the reconstruction of information from past episodic memories.

Schizophrenia (SCZ) is a serious worldwide psychiatric disease that affects about 1% of the general population. Individuals with SCZ are suggested to suffer from disturbances in the sense of continuity of self across time (Danion et al., 2005). Therefore, they may have difficulties in remembering their personal experiences in the past and imagining about the future. Many previous studies have documented that SCZ patients suffer from impairments in episodic memory (e.g., Bilder et al., 2000; Toulopoulou et al., 2003). For example, these patients have been found to recall fewer specific autobiographic memory events (e.g., Riutort et al., 2003; Neumann et al., 2007; Warren and Haslam, 2007). Such an impairment was attributed to the deficits of working self (a subset of working memory) and the consciousness of self identity according to the cognitive-motivation model in the self memory system (Conway and Pleydell-Pearce, 2000). Conway and Pleydell-Pearce (2000) suggested that the ability to generate specific events from the autobiographical memory is related to autonoetic awareness. Due to the close relationship between autobiographical memory and future thinking, the autobiographical memory deficit found in SCZ may also reflect in the future time dimension. This has been examined in several studies. For example, D’Argembeau et al. (2008) and Raffard et al. (2010) found that SCZ patients were impaired in thinking about specific future scenarios. D’Argembeau et al. (2008) also showed that impairment in imagining the future was more severe than in remembering the past. This is consistent with the episodic simulation hypothesis that imagining the future required recombining the retrieved information into novel events, thus it demanded more on flexibility than remembering the past, and SCZ patients were more impaired in future thinking. Furthermore, Corcoran et al. (2006) suggested that anticipating future threatening events in patients with persecutory delusions was significantly predicted by recalling such events. This suggested that reasoning about the future can be predicted by past experiences. Corcoran (2010) suggested that the cognitive deficits (e.g., autobiographical memory, theory of mind, jumping to conclusion) in paranoia could be explained by impairments in mental time travel or cognitive self-projection. All these studies, therefore, demonstrated that mentally travel to the future and past are closely related and mental time travel plays an important role in psychotic disorders.

Most studies on mental time travel focused on its specificity. However, emotion is also very important for mental time travel. SCZ patients have been found to display hedonic capacity deficits such that they cannot anticipate pleasurable activities as found in healthy individuals (Gard et al., 2007). This may be attributed to their limited ability to envision specific details and situations for future events (Strauss and Gold, 2012). This hypothesis is also supported by studies that found SCZ patients to show specificity deficit when asked to recall emotional events (Neumann et al., 2007). Moreover, impaired ability to imagine future pleasant events is associated with apathy in SCZ (Raffard et al., 2013). However, in most of the previous studies, participants generated events with emotional cues (words or pictures), how do SCZ spontaneously generate emotional events (without emotional cues) in mental time travel remained unclear yet. The present study adopted tasks to measure this.

Schizophrenia is now considered as a spectrum including individuals at high risk for developing SCZ. Individuals with schizotypal personality disorder features (SPD proneness) who score high on psychometric tests for schizotypy were among the SCZ spectrum (Cadenhead et al., 1999). These individuals share similar characteristics with SCZ patients, but to a milder degree. Since SCZ patients showed deficits in mental time travel, it is expected that the individuals in the SCZ spectrum may also suffer similar problems. However, to date no study has been conducted to investigate mental time travel in SCZ from a spectrum perspective. Therefore, the present study aimed to explore mental time travel in SCZ spectrum that mainly focuses on individuals with SPD proneness and SCZ patients.

So far, the most widely used laboratory assessment of episodic past and future thinking are the cue-word task (to generate specific events related to a cue word) (Crovitz and Schiffman, 1974; Williams et al., 1996; Addis et al., 2007; Szpunar and McDermott, 2008) and tasks that require participants to generate specific events within a period of time, for example, 1 week, 1 month, and 1 year, (Riutort et al., 2003; Wood and Brewin, 2006). Raes et al. (2007) argued that because of the explicit instruction and the practical examples, the commonly used tasks may not be suitable for assessing episodic past and future thinking especially for subclinical populations. This is because these individuals may only show a mild impairment and providing them with explicit instructions to generate specific events may not be sensitive enough to detect their problems in episodic past and future thinking. To address this issue, Raes et al. (2007) developed the Sentence Completion for Events from the Past Test to evaluate the spontaneous generation of episodic past events. A similar measure for future events, Sentence Completion for Events in the Future Test (SCEFT), was developed by Anderson and Dewhurst (2009). These two instruments were used in the present study to examine past and future thinking in SCZ spectrum.

To summarize, previous studies mainly explored the specificity of episodic past and future thinking in SCZ patients. In the present study we aimed to explore the specificity of past and future thinking in SCZ from a spectrum perspective, and included both individuals with SPD proneness and SCZ patients as participants. We hypothesized that the decrease of specificity may be a marker of SCZ spectrum, and individuals in the SCZ spectrum would generate fewer specific events, particularly future events. Based on the episodic simulation hypothesis, we hypothesized that participants would generate more specific events for the past than for the future, and the specificity of past and future thinking would be correlated. Moreover, we also explored the emotional valence of events generated by these participants and hypothesized that they would generate less positive events compared with the controls.

## Materials and Methods

### Participants

Thirty-two inpatients with SCZ were recruited from psychiatric hospitals (from (D’Argembeau et al., 2008), we take the effect size between SCZ and HC as 1, for power = 0.9 and *p* = 0.05, the sample size needed was 23 for each group calculated by G^∗^power). All of them fulfilled the diagnostic criteria of DSM-IV (American Psychiatric Association, 1994) based on a structured clinical interview (First et al., 1995)^1^. Participants with SPD proneness were screened by the Schizotypal Personality Questionnaire (SPQ) (Raine, 1991; Chen et al., 1997). A total of 303 undergraduate students were recruited and completed the SPQ. According to the manual of SPQ (Raine, 1991), participants who scored in the top 10% of the total sample were selected as the SPD proneness group. The final sample of the study comprised 30 SPD proneness individuals (mean SPQ score = 46.33, *SD* = 5.44). Thirty-three healthy controls (HCs) were recruited from the general community through advertisement (mean SPQ score = 22.87, *SD* = 8.41).

For SPD proneness and HCs, the exclusion criteria were: (1) a personal or family history of neurological illness or alcohol/drug dependence; (2) a personal history of psychiatric disease screened by non-structural interview; (3) HCs with a family history of psychosis. For SCZ patients, those receiving electroconvulsive therapy within the last 3 months or IQ less than 70 were excluded. Their IQ were estimated using the short form (information, arithmetic, similarity, and digit span) of the Chinese version of the Wechsler Adult Intelligence Scale-Revised (WAIS-R) (Gong, 1992). For SCZs, their clinical symptoms were assessed by clinicians with the Positive and Negative Syndrome Scale (PANSS, Kay et al., 1987). All patients were treated with atypical antipsychotics (e.g., Risperidone). Abnormal Involuntary Movements Scale (AIMS, Smith et al., 1979) and Barnes Akathisia Rating Scale (BARS, Barnes, 1989) were used to evaluate side effects of the medication. Demographic and clinical information of all participants are summarized in **Table 1**. Since gender, age, IQ were not matched among groups, they were controlled as covariates in the data analyses. The current study was approved by the ethics committee of Institute of Psychology, Chinese Academy of Sciences and Mental Health Hospitals. All participants were given a brief introduction of the study and an opportunity to ask questions. All of them provided written informed consent before the commencement of the study and were paid 50 RMB for their participation.

**Table 1:** Demographic information of participants.

	Control (*n* = 33)		Schizophrenia (*n* = 32)		SPD proneness (*n* = 30)		*F/χ^2^*	*p*
					
	Mean	*SD*	Mean	*SD*	Mean	*SD*		
Male: female	20: 13		19: 13		8: 22		9.13	0.010
Age	28.09	9.39	34.75	9.23	19.96	1.75	26.49	<0.001
Education	13.12	2.64	12.19	3.01	13.58	1.19	2.67	0.075
IQ	119.94	15.68	106.66	12.27	124.23	10.71	15.30	<0.001
SPQ score	22.87	8.41	∖	∖	46.33	5.44	164.63	<0.001
Duration of illness (years)			7.66	4.89				
Medication (chlopromazine equivalence mg/day)			331.29	212.63				
PANSS								
Positive symptoms			11.16	3.71				
Negative symptoms			12.61	4.09				
General psychopathology			23.29	6.81				
Total score			47.06	12.81				
AIMS			1.00	1.78				
BARS			0.90	1.64				


*PANSS, Positive and Negative Syndrome Scale; AIMS, Abnormal Involuntary Movements Scale; BARS, Barnes Akathisia Rating Scale.*

### Materials

#### Sentence Completion for Events from the Past Test (SCEPT)

The SCEPT (Raes et al., 2007) has 11 sentence stems for participants to generate their past experiences. For example, “I still remember well how …”. Participants were required to complete the sentence stems in any forms, but they were instructed that the contents must be on different topics for each of these sentences. The completed sentences were rated for three aspects: specificity, emotional valence and content. Specificity has five categories: specific (a specific event happening at a particular time and place within a day), extended (a specific event lasting for more than 1 day), categorical (a general event belonging to a category), semantic associates (semantic information), and omission (participant cannot recall anything) (Raes et al., 2007; Anderson and Dewhurst, 2009). The emotional valence of the sentences was coded into positive, negative and neutral according to the events generated by the participants.

#### Sentence Completion for Events in the Future Test

The SCEFT (Anderson and Dewhurst, 2009) also has 11 sentence stems for participants to generate possible events for the future For example, “ln the future I can imagine well how …”. The requirements for the test were the same as the SCEFT in that participants can finish the sentences in any way they want, but the contents must be on different topics for each of the sentences. The rating of the SCEFT is the same as the SCEPT. The Chinese version of SCEPT and SCEFT (Chen et al., 2015) were used in this study. The order of SCEPT and SCEFT were counterbalanced across participants.

### Scoring and Statistical Analyses

Two raters coded the answers for the SCEPT and SCEFT according to the scoring criteria for specificity and emotion valence). They were blind to the diagnosis of the participants. For items where the raters did not agree, their scores were discussed and reconciled with a third rater. The proportions of events generated in each category in each aspect of the ratings were the main measures of the participants’ performance. The inter-rater reliabilities (calculated with Cohen’s Kappa) are good (specificity: *K* = 0.81; emotional valence: *K* = 0.85) for these two tasks.

Data analyses were conducted on two aspects of the SCEPT and SCEFT. For specificity, we conducted a 3(Group: SPD proneness, SCZ, HCs) × 2(Time orientation: past, future) ANCOVA on each of the four categories of specificity (specific, extended, categorical, and semantic associates). For emotional valence, a 3(Group: SPD proneness, SCZ, HCs) × 2(Time orientation: past, future) ANCOVA was conducted on different emotional valence of the generated events. Normality test revealed that these dependant variables were all near normal distribution (Skewness and Kurtosis less than 1).

A number of Pearson correlational analyses were also conducted: first, the correlation between the proportion of specific past experiences and the proportion of specific future imagination was calculated. Second, the relationships between clinical symptoms and performances on the sentence completion were analyzed.

## Results

The mean proportions of each category in the two rating aspects are displayed in **Table 2**.

**Table 2:** Mean (SD) proportions of the different response categories across task type in participants.

	Control		Schizophrenia		SPD proneness		Group	Time orientation	Interaction
						
	SCEPT	SCEFT	SCEPT	SCEFT	SCEPT	SCEFT	*p*	*p*	*p*
**Specificity**								
Specific events	0.340 (0.170)	0.268 (0.134)	0.307 (0.140)	0.171 (0.122)	0.352 (0.173)	0.200 (0.144)	0.017	ns	0.03
Extended events	0.298 (0.153)	0.240 (0.149)	0.148 (0.124)	0.125 (0.128)	0.130 (0.116)	0.164 (0.150)	<0.001	ns	ns
Categoric events	0.130 (0.094)	0.278 (0.114)	0.190 (0.112)	0.253 (0.122)	0.239 (0.142)	0.476 (0.192)	<0.001	ns	0.04
Semantic associates	0.242 (0.128)	0.209 (0.119)	0.338 (0.146)	0.418 (0.168)	0.276 (0.203)	0.158 (0.155)	0.004	ns	<0.001
**Emotion**									
Positive	0.300 (0.163)	0.471 (0.165)	0.210 (0.129)	0.367 (0.190)	0.209 (0.153)	0.336 (0.174)	0.001	ns	ns
Neutral	0.499 (0.182)	0.499 (0.158)	0.585 (0.165)	0.548 (0.185)	0.582 (0.217)	0.627 (0.169)	0.004	ns	ns
Negative	0.201 (0.155)	0.030 (0.054)	0.188 (0.150)	0.051 (0.138)	0.206 (0.149)	0.033 (0.077)	ns	ns	ns


*SCEPF, Sentence Completion for Events from the Past Test; SCEFT, Sentence Completion for Events from the Future Test. ns, non-significant.*

### Specificity

For specific events, the main effect of Group was significant, *F*(2,89) = 4.27, *MSE* = 0.030, *p* = 0.017. Pairwise comparisons showed that HCs (*M* = 0.308)^2^ generated more specific events than SCZ (*M* = 0.208) (*p* = 0.015, Bonferroni corrected). There was no significant difference between SPD proneness individuals (*M* = 0.302) and HCs, or between SPD proneness individuals and SCZ (both *p*s > 0.05, Bonferroni corrected). The main effect of Time orientation was not significant (*p* > 0.05)^3^. The interaction between Group and Time orientation was significant, *F*(2,89) = 3.65, *MSE* = 0.012, *p* = 0.03. Simple effect analyses showed that when recalling the past, there was no significant difference among HCs, SPD proneness individuals, and SCZ (all *p*s > 0.05, Bonferroni corrected). When imagining the future, both HCs and SPD proneness individuals imagined more specific events than SCZ (HCs > SCZ: *p* < 0.001; SPD > SCZ: *p* = 0.016; Bonferroni corrected); there was no significant difference between HCs and SPD proneness individuals (*p* > 0.05, Bonferroni corrected).

For extended events, the main effect of Group was significant, *F*(2,89) = 15.58, *MSE* = 0.023, *p* < 0.001. Pairwise comparisons showed that HCs (*M* = 0.266) generated more extended events than SPD proneness individuals (*M* = 0.102) (*p* < 0.001, Bonferroni corrected) and SCZ (*M* = 0.182) (*p* = 0.021, Bonferroni corrected); there was no significant difference between SPD proneness individuals and SCZ (*p* > 0.05, Bonferroni corrected). The main effect of Time orientation and interaction between Group and Time orientation were not significant (*p*s > 0.05)^4^.

For categoric events, the main effect of Group was significant, *F*(2,89) = 11.71, *MSE* = 0.022, *p* < 0.001. Pairwise comparisons showed that HCs (*M* = 0.204) and SCZ (*M* = 0.229) generated less categoric events than SPD proneness individuals (*M* = 0.350) (HCs < SPD, *p* < 0.001; SCZ < SPD, *p* = 0.006, Bonferroni corrected); there was no significant difference between HCs and SCZ (*p* > 0.05, Bonferroni corrected). The main effect of Time orientation was not significant (*p* > 0.05)^5^. The interaction between Group and Time orientation was significant, *F*(2,89) = 3.33, *MSE* = 0.013, *p* = 0.04. Simple effect analyses showed that when recalling the past, HCs generated less categoric events than SPD proneness individuals (*p* = 0.004, Bonferroni corrected); there was no significant differences between HCs and SCZ or between SCZ and SPD proneness individuals (both *p*s > 0.05, Bonferroni corrected). When imagining the future, SPD proneness individuals generated more categoric events than HCs and SCZ (both *p*s < 0.001, Bonferroni corrected), HCs and SCZ did not show significant difference (*p* > 0.05).

For semantic associated events, the main effect of Group was significant, *F*(2,89) = 5.94, *MSE* = 0.036, *p* = 0.004. Pairwise comparisons showed that HCs (*M* = 0.223) generated significantly less semantic associates than SCZ (*M* = 0.354) (*p* = 0.003, Bonferroni corrected), there was no significant difference between HCs and SPD proneness individuals (*M* = 0.245), or between SPD proneness individuals and SCZ (both *p*s > 0.05). The main effect of Time orientation was not significant (*p* > 0.05)^6^. The interaction between Group and Time orientation was significant, *F*(2,89) = 8.96, *MSE* = 0.012, *p* < 0.001. Simple effect analyses showed that when recalling the past, there was no significant differences among the three groups of participants (all *p*s > 0.05, Bonferroni corrected). When imagining the future, both HCs and SPD proneness individuals imagined less semantic associates than SCZ (both *p*s < 0.001, Bonferroni corrected); there was no significant difference between HCs and SPD proneness individuals (*p* > 0.05, Bonferroni corrected).

### Emotional Valence

For the positive events, the main effect of Group was significant [*F*(2,89) = 7.33, *MSE* = 0.032, *p* = 0.001]. Pairwise comparisons suggested that HCs generated more positive events compared with SCZ (*p* = 0.04, Bonferroni corrected) and SPD proneness individuals (*p* = 0.004, Bonferroni corrected); there was no significant difference between SPD proneness individuals and SCZ (*p* > 0.05, Bonferroni corrected). The main effect of Time orientation [*F*(2,89) = 0.57, *MSE* = 0.022, *p* = 0.453] and the interaction between Group and Time orientation [*F*(2,89) = 1.15, *MSE* = 0.022, *p* = 0.322] were not significant^7^. As to the neutral events, the main effect of Group was significant [*F*(2,89) = 5.96, *MSE* = 0.040, *p* = 0.004]. Pairwise comparisons suggested that SPD proneness participants generated more neutral events than HCs (*p* = 0.003, Bonferroni corrected); the difference between HCs and SCZ or between SPD proneness individuals and SCZ were not significant (both *p*s > 0.05, Bonferroni corrected). The main effect of Time orientation [*F*(1,89) = 0.30, *MSE* = 0.025, *p* = 0.583] and the interaction between Group and Time orientation [*F*(2,89) = 0.94, *MSE* = 0.025, *p* = 0.395] were not significant^8^. For the negative events, the main effect of Group [*F*(2,89) = 0.55, *MSE* = 0.020, *p* = 0.580], the main effect of Time orientation [*F*(2,89) = 0.19, *MSE* = 0.012, *p* = 0.668] and the interaction [*F*(2,89) = 0.10, *MSE* = 0.012, *p* = 0.905] were not significant^9^.

### Correlational Analyses

The correlation between the proportions of the specific past and future events was significant (*r* = 0.416, *p* < 0.001). For correlations between clinical symptoms and specificity of past and future thinking in SCZ patients, there was a significant negative correlation between general psychopathology and proportion of past specific events (*r* = -0.532, *p* < 0.01). No other significant correlations were found.

## Discussion

Schizophrenia is a spectrum of disorders. Exploring SCZ from a spectrum perspective will help us to identify biological markers of the disease for early detection and intervention. To our knowledge, this study explored mental time travel from a spectrum perspective for the first time. For specificity of mental time travel, individuals with SPD proneness were only impaired in generating extended events in the current study. To date, there was only one study on mental time travel in this population (Winfield and Kamboj, 2010). However, that study focused on phenomenological characteristics while we focused on specificity and emotional valence. The SPD proneness participants showed deficits only in the extended events but not specific events. The reason may be that the SPD proneness participants are good functional college students. Therefore, their impairments were not that severe and they did not show deficits in specific events but only showed deficits in extended events.

Schizophrenia showed deficits in specific events. For future thinking, SCZ generated significantly less specific events than controls, and this finding is consistent with those reported in previous studies (D’Argembeau et al., 2008; Raffard et al., 2013). According to the episodic simulation hypothesis, episodic memory plays a very important role in episodic future thinking. People have to reconstruct the information, details in their episodic memory when imagine the future (Schacter and Addis, 2007a,b). During this process, working memory is necessary to maintain the specific information and details about the past events as well as to make the reconstruction (Warren and Haslam, 2007). SCZ suffer from general cognitive deficits including attention, working memory etc. (Fioravanti et al., 2005). Moreover, SCZ is a self-disorder, and they suffered from disturbances in the sense of continuity of self across time (Danion et al., 2005). This is also consistent with previous findings that SCZ patients were impaired in autonoetic awareness for the future (de Oliveira et al., 2009). Thus, the deficits of specificity for future thinking may be related to their impairments in working memory and self impairment. Further studies are needed to explore the underlying mechanisms. The fact that participants generated more specific events for the past than for the future and the proportion of specific events generated for the past and future were significantly correlated provided support for the episodic simulation hypothesis from the SCZ spectrum populations.

However, in the current study SCZ did not show any deficit in recalling specific past events which is not consistent with most previous studies (Riutort et al., 2003; Danion et al., 2005; McLeod et al., 2006; Neumann et al., 2007; D’Argembeau et al., 2008). The relatively intact ability to recall specific events in SCZ may be because in previous studies, the explicit instructions provided helped healthy controls to generate more specific experiences especially for the past. However, SCZ may not use the explicit tips to generate specific events as well as the healthy participants. In the present study, the assessments measured spontaneous past and future thinking without explicit instructions. As a result, the difference between SCZ and healthy controls were diminished. This explanation is supported by the fact that the proportion of specific events generated in the present study (less than 0.4) was lower than previous findings (larger than 0.8, D’Argembeau et al., 2008) in HCs and the null findings in another study that did not use explicit instructions for specificity (Raffard et al., 2009). From the spectrum perspective, the SCZ showed significant impairment in generating specific events, while SPD proneness individuals did not show impairment in generating specific events but only generating less extended events, indicating that the severity of impairment in mental time travel varies across the spectrum, with SCZ patients showed most severe deficits.

For emotional valence about the events, both individuals with SPD proneness and SCZ shared similar patterns in that they were less positive compared with HCs. And there was no significant difference between SCZ and SPD proneness individuals. Previous studies suggested that people usually have a positivity bias in future thinking since human usually have uncorrected positive future illusions (Finnbogadottir and Berntsen, 2013). Also, future plans are always related to personal goals and achievements (Boyer, 2008), thus people would like to anticipate positive results. Although SPD individuals recalled more positive events than negative events, they imagined comparable positive and negative events for the future. Moreover, SPD individuals generated less positive events than controls regardless whether it was recalling the past or imagining the future. Previous studies suggested that individuals with SPD proneness exhibited deficient anticipatory pleasure (Yan et al., 2011; Shi et al., 2012). Thus, they may imagine fewer positive events in the future.

For SCZ, similar findings were reported in Raffard et al.’s (2013) study that they imagined less future positive events which was associated with their apathy. Our results showed that SCZ generated less positive events irrespective of the past and future. This could be because SCZ have more negative self-beliefs (Green et al., 2012) and reduced optimism (Prentice et al., 2005). Moreover, SCZ have been reported to have problems in hedonic capacity (Strauss and Gold, 2012). They cannot enjoy and anticipate pleasant experiences. SCZ also had a reduced positive bias for non-current feelings (Raffard et al., 2013). All these would contribute to the less amount of positive events they generated.

### Limitations and Future Directions

There are several limitations in the present study. First, we only focused on two groups of people in the SCZ spectrum disorders. Other groups like the first-degree relatives of SCZ were not included. To understand episodic past and future thinking in this spectrum more comprehensively, further studies are needed to include other populations (such as first-degree relatives of SCZ, ultra high risk cases). Second, the Axis II disorders were not screened in SPD proneness and HCs, this might have affected the results, further studies should screen for Axis II disorders. Third, the present study only described the extent of impairments in past and future thinking in SCZ spectrum disorders. However, we did not attempt to investigate the underlying cognitive and neural mechanisms of these impairments. Further studies are, therefore, needed to provide a comprehensive picture of the cognitive and neural mechanisms of past and future thinking in this population. Fourth, due to the task we used in the present study, we could not analyze the details and phenomenological characteristics of the events generated by participants, further studies can use other tasks to explore these issues.

## Conclusion

The current study examined mental time travel in the SCZ spectrum disorders. Results showed that individuals with SPD proneness and SCZ patients showed problems in generating specific events about the past and future. SPD proneness individuals showed mild problems only in generating extended events. SCZ patients had more serious problems in imagining specific events, recalling and imagining extended events which indicates the decrease of specificity of past and future thinking may be a marker for SCZ. For emotional valence, both SPD proneness individuals and SCZ patients generated less positive events.

## Author Contributions

X-jC and YW designed the study, X-jC, L-lL, and F-hL collected the data, H-fZ, M-yG, and C-qL help recruited the clinical cases and made the clinical ratings, X-jC, L-lL, and YW wrote up the first draft, J-fC, A-tC, W-hW, DS, and RC provided insightful comments to the manuscript and help revised the manuscript, all authors proved the final version of the manuscript.

## Conflict of Interest Statement

The authors declare that the research was conducted in the absence of any commercial or financial relationships that could be construed as a potential conflict of interest.

## Footnotes

1 The structured interview was administered by the doctor in charge of the patient and the diagnosis was confirmed by an upper level psychiatrist.

2 The means in the parentheses are estimated marginal means when covariates are taken into consideration.

3 The effect of covariates: Gender, *F*(1,89) = 1.36, *MSE* = 0.030, *p* = 0.247; Age, *F*(1,89) = 3.90, *MSE* = 0.030, *p* = 0.051; IQ, *F*(1,89) = 1.05, *MSE* = 0.030, *p* = 0.309.

4 The effect of covariates: Gender, *F*(1,89) = 0.00, *MSE* = 0.023, *p* = 0.972; Age, *F*(1,89) = 8.86, *MSE* = 0.023, *p* = 0.004; IQ, *F*(1,89) = 2.55, *MSE* = 0.023, *p* = 0.114.

5 The effect of covariates: Gender, *F*(1,89) = 1.27, *MSE* = 0.022, *p* = 0.263; Age, *F*(1,89) = 0.28, *MSE* = 0.022, *p* = 0.598; IQ, *F*(1,89) = 1.25, *MSE* = 0.022, *p* = 0.268.

6 The effect of covariates: Gender, *F*(1,89) = 3.06, *MSE* = 0.036, *p* = 0.084; Age, *F*(1,89) = 0.30, *MSE* = 0.036, *p* = 0.588; IQ, *F*(1,89) = 1.06, *MSE* = 0.036, *p* = 0.305.

7 The effect of covairates: Gender, *F*(1,89) = 0.01, *MSE* = 0.032, *p* = 0.946; Age, *F*(1,89) = 0.25, *MSE* = 0.032, *p* = 0.622; IQ, *F*(1,89) = 0.01, *MSE* = 0.032, *p* = 0.925.

8 The effect of covariates: Gender, *F*(1,89) = 0.08, *MSE* = 0.040, *p* = 0.777; Age, *F*(1,89) = 3.54, *MSE* = 0.040, *p* = 0.063; IQ, *F*(1,89) = 0.00, *MSE* = 0.040, *p* = 0.997.

9 The effect of covariates: Gender, *F*(1,89) = 1.19, *MSE* = 0.020, *p* = 0.543; Age, *F*(1,89) = 2.36, *MSE* = 0.020, *p* = 0.128; IQ, *F*(1,89) = 0.03, *MSE* = 0.020, *p* = 0.874.
